# Prevalence, Mechanisms, and Implications of Gastrointestinal Symptoms in COVID-19

**DOI:** 10.3389/fmed.2020.588711

**Published:** 2020-10-30

**Authors:** Abhilash Perisetti, Hemant Goyal, Mahesh Gajendran, Umesha Boregowda, Rupinder Mann, Neil Sharma

**Affiliations:** ^1^Department of Gastroenterology and Hepatology, University of Arkansas for Medical Sciences, Little Rock, AR, United States; ^2^The Wright Center for Graduate Medical Education, Scranton, PA, United States; ^3^Department of Internal Medicine, Mercer University School of Medicine, Macon, GA, United States; ^4^Paul L. Foster School of Medicine, Texas Tech University Health Sciences Center El Paso, El Paso, TX, United States; ^5^Department of Internal Medicine, Bassett Medical Center, Cooperstown, NY, United States; ^6^Saint Agnes Medical Center, Academic Hospitalists, Fresno, CA, United States; ^7^Division of Interventional Oncology and Surgical Endoscopy (IOSE), Parkview Cancer Institute, Fort Wayne, IN, United States; ^8^Division of Interventional Oncology and Surgical Endoscopy, Indiana University School of Medicine, Fort Wayne, IN, United States

**Keywords:** COVID-19, SARS-CoV-2, endoscopy, gastrointestinal symptoms, diarrhea, fecal-oral transmission

## Abstract

Coronavirus disease 2019 (COVID-19) is caused by severe acute respiratory syndrome coronavirus-2 (SARS-CoV-2) infection. The infection started as an outbreak of pneumonia-like symptoms in Wuhan, China. Within a few weeks, it spread across the entire globe resulting in millions of cases and thousands of deaths. While respiratory symptoms and complications are well-defined and can be severe, non-respiratory symptoms of COVID-19 are increasingly being recognized. Gastrointestinal manifestations such as nausea, vomiting, diarrhea, and abdominal pain have been added to the list of common COVID-19 symptoms. Their prevalence has been increasing, probably due to increased recognition and experience with the pandemic. Furthermore, diarrhea and stool testing may change prevalence and transmission rates due to suspicion for fecal-oral transmission of the COVID-19. Due to this risk, various countries have started testing wastewater and sewage systems to examine its role in the spread of SARS-CoV-2 among communities. In this review article, we describe the common gastrointestinal manifestations in COVID-19, their prevalence based upon the current literature, and highlight the importance of early recognition and prompt attention. We also note the role of fecal-oral transmission. Furthermore, the mechanisms of these symptoms, the role of medications, and potential contributing factors are also elaborated.

## Introduction

Coronavirus disease 2019 (COVID-19) is caused by severe acute respiratory syndrome coronavirus 2 (SARS-CoV-2) manifesting mainly as pneumonia, acute respiratory distress syndrome, and multiorgan failure (1). Though originated in Wuhan, China, as a cluster of pneumonia-like presentation, soon it spread across the globe. As of July 28th, 2020, SARS-CoV-2 has affected all countries and territories in the World, with more than 17 million cases and 660,000 deaths (2). Non-respiratory manifestations are increasingly being recognized in COVID-19 (3, 4). GI symptoms though less common compared to respiratory symptoms have gained increased significance lately (5). The Center for Disease Control (CDC) added multiple GI symptoms of COVID-19, which can coexist with respiratory symptoms, or they can be the only presentation of the disease (3). Anorexia, nausea, vomiting, diarrhea, and abdominal pain are some of the frequently observed gastrointestinal (GI) symptoms in COVID-19 patients (6). Additionally, GI bleeding, acute pancreatitis, and colitis have also been reported.

There is a potential of fecal-oral transmission due to the presence of the viral RNA in stools, especially in asymptomatic individuals. Due to this concern, sewage and the wastewater is being analyzed to detect the SARS-CoV-2 virus, examine the role of feco-oral transmission in the community, and identify mitigation strategies (7). Early identification and prompt attention to GI symptoms are critical because hospitalized COVID-19 patients with concomitant GI symptoms have been found to develop severe disease. Also, understanding the prevalence and mechanisms of GI manifestations can help to better characterization of these symptoms. In addition, educating patients about these symptoms can not only identify COVID-19 cases at an early stage but also can prevent potential spread to uninfected individuals.

## Anorexia

Loss of appetite (anorexia) is one of the most common GI symptoms in COVID-19 patients (Table 1). The presence of anorexia in COVID19 is mostly underestimated and under-reported because of its non-specific nature. Prevalence among different studies ranged from 12.2 to 50.2% (8–10). A pooled analysis of multiple studies showed an overall prevalence of 26.8% (11). Anorexia is also frequently associated with other GI symptoms of vomiting, abdominal pain, and diarrhea. Pathophysiology of anorexia could be related to acute viral prodrome associated with COVID-19. Acute inflammation can increase the cytokine load such interleukins (IL-2, IL-7), tumor necrosis factors (TNF) which contribute to cytokine storm seen in COVID-19 (Table 2). In addition, altered or change in taste (dysgeusia) noted in these patients can further exacerbate loss of appetite (26).

**Table 1:** Typical and atypical GI symptoms in COVID-19.

**Typical**	**Atypical**
Loss of appetite (anorexia)	Altered taste (dysgeusia)
Nausea and vomiting	Gastrointestinal bleeding
Diarrhea	Secondary bacterial infection (*Clostridium difficile*)
Abdominal pain	

**Table 2:** Mechanisms of GI symptoms.

**Effect**	**Contributing factors**
Viral cytopathic effect	• The entry of SARS-CoV-2 via ACE-2 receptors in GI glandular epithelium (12) • Isolated of viral RNA particles in stools of COVID-19 patients (13)
Altered gut microbiota	• Use of multiple antimicrobial agents • Change in gut microbial composition from viral proinflammatory mediators (14) • Abnormal mTOR activity and decreased antimicrobial activity (15) • Increased susceptibility for infections (*Clostridium difficile*) (16) • Hypochlorhydria induced by antisecretory agents (such as the use of proton pump inhibitors) (17) • Altered gut-lung axis (18)
Inflammation	• Increased cytokine release such as interleukins (IL-2, 7), tumor necrosis factor, granulocyte monocyte colony-stimulating factors (cytokine storm) (19) • The altered gut-brain axis (20) • Increased fecal calprotectin (21)
Worsening of prior GI conditions	• Overexpression of ACE-2 in the inflamed gut in inflammatory bowel disease (21) • Worsening of prior irritable bowel syndrome
Secondary infections	• Increased risk of *clostridium difficile* (16)
Others	• Intestinal ischemia (22, 23) • Viral colitis (24) • Altered GI epithelial integrity (25) • Altered enteric nervous system output (20)

*SARS-CoV-2, Severe acute respiratory syndrome coronavirus 2; ACE-2, angiotensin-converting enzyme-2*.

## Nausea and Vomiting

COVID-19 patients can have nausea and vomiting as their only symptoms at presentation or as a combination with other GI symptoms, including anorexia, diarrhea, and rarely abdominal pain. A pooled prevalence of 7.8% (95% CI, 7.1–8.5%) was noted for nausea or vomiting from 26 studies spanning among different nations. Similar to other GI symptoms, the prevalence of nausea and vomiting among Chinese studies was lower (5.2%) compared to studies from other countries (14.9%). In one study' s Nobel et al. (27) noted that the presence of nausea or vomiting in as high as 22.7% patients (63 patients developed nausea among 278 patients, 95% CI, 17.9–28%). Similarly, Cholankeril et al. (28) and Hajifathalian et al. (29) reported the prevalence of symptoms of nausea and vomiting as 10.3 and 15.9%, respectively. While the precise reasons remain unclear, nausea, and vomiting could be related to a combination of effects on the gut and central nervous system (CNS) (13). After entry of SARS-CoV-2 into the GI tract, it can gain access to portal circulation and can affect the vagus nerve either through vascular or lymphatic routes. In addition, the cytopathic effect caused by SARS-CoV-2 combined with cytokine storm can stimulate central and peripheral (autonomic nervous) pathways, culminates into a sensation of nausea (with or without vomiting). Once neural pathways are stimulated, gastric dysrhythmia can occur, resulting in vomiting (30). Furthermore, antibiotics and antiviral agents are frequently used in COVID-19 patients, which further exacerbates their symptoms (31). If these factors contribute in an isolated fashion or combination is unknown.

## Diarrhea

Diarrhea is a commonly noted GI symptom in COVID-19 patients. It has significant public health importance given its potential for feco-oral transmission of disease. A pooled prevalence of multiple studies showed an overall prevalence of diarrhea of 5–10% (11, 32). There is a wide range of prevalence noted in multiple studies ranging from 2 to 50% (33). In a large cohort of 1,059 patients, 234 cases of diarrhea were noted with a prevalence of 22.1% (95% CI, 19.6–24.7%) (29). Similarly, among 355 cases in the Hubei province of China, 130 patients developed diarrhea with a prevalence of 36.6% (95% CI, 31.6–41.9%) (34). Several factors could be responsible for variation in the prevalence of diarrhea in these studies. Documentation of GI symptoms at the time of hospitalization, high suspicion, and early recognition, and if patients are treated either in an outpatient or inpatient basis, could be responsible for this variation.

Despite the high frequency of diarrhea, standardized criteria for diagnosis, and grading the severity of diarrhea are missing in most studies. Patients with a viral illness can present with a transient episode of loose stools with or without other GI symptoms. While persistent diarrhea (3 or more loose stools for more than 48 h) is significant, this definition is rarely used in the studies. Moreover, if diarrhea is not present at the admission, it becomes challenging to ascertain the cause of diarrhea. Several confounding variables, such as the use of enteral feeding (tube feeds), antiviral and antibiotics, altered gut flora, hyperinflammatory response, secondary bacterial infections, use of proton pump inhibitors (PPI) can potentially cause diarrhea in hospitalized COVID-19 patients (35). In order to determine if the persistent diarrhea is from SARS-CoV-2, evidence of direct viral-induced cytopathy (through histology or stool viral RNA positivity) should be documented.

## Abdominal Pain

Patients with COVID-19 can present with abdominal pain, which is less frequent as compared to anorexia, nausea/vomiting, or diarrhea. The prevalence of abdominal pain ranges from 3.9 to 6.8% (10, 11, 28, 29, 36). There is no consensus regarding the severity and duration of abdominal pain in COVID-19 patients. The location of the pain could be the right upper quadrant or epigastric or generalized. Few cases of COVID-19 presenting as acute abdomen has been reported (37). The precise mechanism for abdominal pain is unclear. Furthermore, any viral illness as a part of the prodrome can cause transient abdominal cramping and discomfort. Furthermore, abdominal pain can be combined with other GI symptoms of anorexia, nausea with or without vomiting. After the entry into GI tract, SARS-CoV-2 can exert its cytopathic/inflammatory changes, which can potentially lead to visceral pain. If this is a somatic due to the involvement of the peritoneum or a referred pain is unknown. Sporadic reports of pancreatitis have also been reported in COVID-19 patients (38). Additionally, high expression of ACE-2 receptors is noted in the pancreatic tissue, which makes it susceptible to its cytopathic effects. It can lead to leakage of pancreatic lipase and fatty acid oxidation. Few autopsy reports have shown ongoing pancreatic injury in COVID-19 patients without clinically evident acute pancreatitis (18). Hyperlipasemia has been identified in these patients in multiple studies (39, 40). It is unclear if a low level of elevated lipase is from viral pancreatic inflammation or as a part of viral gastroenteritis (38, 39).

## Additional Digestive Symptoms

In addition to the above GI symptoms, other atypical manifestations such as changes or loss of smell and taste and GI bleeding have been documented in COVID-19 patients (17, 41). Aziz et al. noted that taste changes (ageusia/dysgeusia) are prevalent in up to 49.8% (95% CI: 8.2–91.5%, *I*^2^ = 99.6%) patients, although this meta-analysis had limited number of studies (26). Lin et al. reported a few cases of GI bleeding with viral RNA isolation from esophageal samples (12). Furthermore, endoscopically herpetic erosion was noted in these patients. If SARS-CoV-2 cytopathic effects lead to these erosions or if the virus is a bystander remains unclear. Secondary bacterial infections such as *Clostridium difficile* infections were noted in COVID-19 patients, probably due to the widespread use of antibiotics in these patients. Additionally, altered gut flora is documented in these patients, making them vulnerable to these infections (14).

## Putative Mechanisms of Gastrointestinal Symptoms

SARS-CoV-2 enters the mucous membranes (nose, oral cavity) through its well-documented functional receptor angiotensin-converting enzyme-2 (ACE-2) (15, 26). While it can make its way to the gastric lumen via salivary secretions, it is subjected to the adverse effect of the acidic environment of the stomach. A pH of <2 significantly affects the life of the virus (16, 19). Patients with hypochlorhydria are susceptible to get a viral infection because of a higher viral load entering the small intestine (20). ACE-2 receptor concentrations differ among different GI tissues, with high expression noted in ileal enterocytes (21). Once SARS-CoV-2 enters the enterocytes, viral synthesis, replication can continue, and a cytopathic effect is noted (evidenced by intracellular staining of viral nucleocapsid) (22). The virus can continue its journey from here to other organs via the portal circulation. These changes can potentially lead to stool viral RNA positivity. If the presence of viral RNA in the stool is indicative of cytopathic changes or just a bystander needs further validation (Table 2).

Gut flora plays a significant role in maintaining GI homeostasis, and any perturbations can lead to diarrhea and various GI symptoms such as nausea and vomiting. COVID-19 patients are at high risk of microbiome alterations. We highlight 6 of the key factors for gut flora alteration in COVID patients. First, viral infections can increase the release of proinflammatory cytokines, which can alter gut flora (23). It is well-recognized that SARS-CoV-2 patients have elevated cytokines and markers of inflammation (24). Additionally, the use of various antimicrobial medications (antibiotics, antivirals) can change the composition of flora, which can predispose individuals to GI adverse effects (31). A third factor, as respiratory symptoms are exceedingly common in COVID-19 patients, change in lung flora can contribute to the potential change in gut flora (14). This “gut-lung” axis is increasingly being recognized as a potential cause of GI symptoms in individuals with respiratory manifestations (14). A fourth factor is that altered flora can lead to a change in the ratio of pathogenic organisms, potentially leading to infections such as clostridium difficile. Recent reports of such infections were noted in COVID-19 patients (25). Fifth, the use of enteral nutrition, such as tube feeds, can further alter the gut microbiome already affected due to the aforementioned causes. Finally, ACE-2 receptor binding has been shown to alter flora by its aberrant mTOR activity (42).

**Figure 1:**
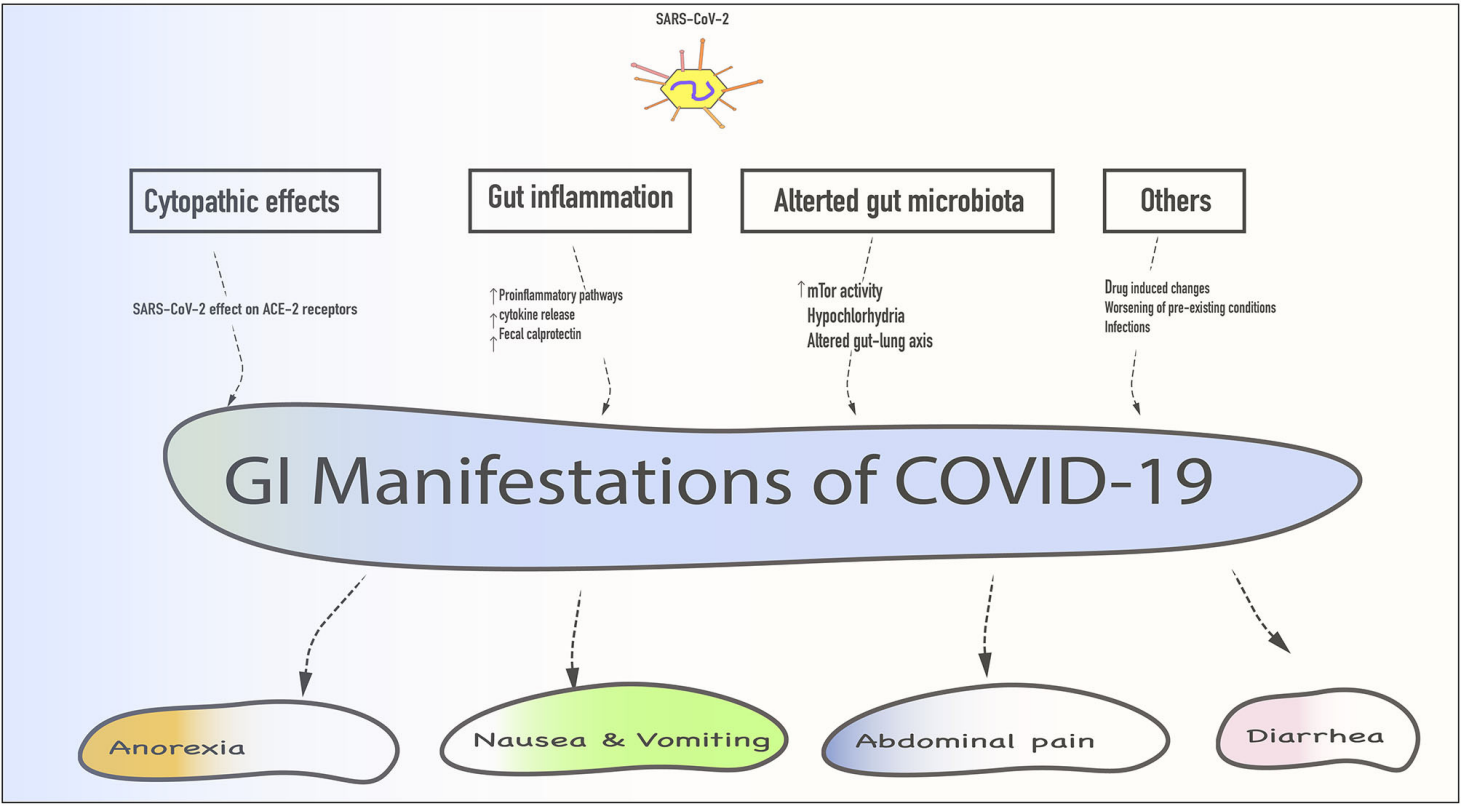
Schematic representation of various gastrointestinal (GI) manifestations with their mechanisms. SARS-CoV-2 gains entry via mucous membranes of the oral cavity enters the stomach and small intestine to exert its cytopathic effect. Additionally, gut inflammation, altered gut flora, drug-induced changes, worsening of pre-existing GI condition, and secondary infections could contribute to these symptoms.

All of the above mechanisms are only putative, as there is no reliable evidence if these mechanisms play a role independently or in combination in the development of GI symptoms in COVID-19 patients. Patients with pre-existing GI conditions such as inflammatory bowel disease (IBD) and malabsorption syndromes etc. are at risk of worsening GI symptoms if infected with SARS-CoV-2 (43, 44). While changes in the gut flora are universal in these populations, ACE-2 expression is elevated in IBD and inflammatory states (44). Fecal calprotectin, which is a marker for bowel inflammation, has been noted to be elevated in COVID-19 individuals with persistent diarrhea (45). If such increased expression predisposes these individuals to worsening symptoms needs to be studied. Furthermore, the enteric nervous system is integrally associated with GI motility, and any perturbation in these pathways can lead to worsening of GI symptoms (46) (Figure 1).

## GI Manifestations and COVID-19 Severity

As noted above, patients with COVID-19 frequently present with GI manifestations. However, data on the correlation between GI manifestations and severity of COVID-19 has been variable (6, 47–50). Ramachandran et al. studied 150 hospitalized patients with COVID-19 with and without GI symptoms and reported no difference in length of stay or need for mechanical ventilation or mortality (6). Pan et al. reported that as the severity of COVID-19 increased, GI symptoms were more pronounced (47). A pooled analysis of multiple studies evaluated the correlation between GI symptoms and COVID-19 severity. Abdominal pain was associated with 4 fold increased odds of severe disease, marginally increased odds with nausea/vomiting, and no correction with diarrhea (48). Further dedicated studies are needed to evaluate these correlations of GI symptoms and COVID-19 severity without potential confounders.

## Fecal-Oral Spread

Diarrhea can predispose the community to the fecal-oral spread of the disease. Previous outbreaks from other coronaviruses (SARS outbreak in 2003) showed that sewage could be a source of infection (51). Randazzo et al. noted that evaluating waste-water plant systems early in the outbreak can help in identifying the spread of infection even before patients exhibit clinical symptoms (7). These methods can potentially help in developing public health strategies and policies to interfere with the spread of the disease.

In patients with diarrhea, stool positivity is noted in almost half of the cases. A systematic review of multiple studies showed a pooled prevalence of 48.1% (95% CI, 38.3–57.9%) for stool positivity (11). It is debatable if this stool positivity can lead to infectivity and spread of disease to uninfected individuals. COVID-19 Patients can have viral RNA stools positivity for an extended period (up to 14 days) even after the resolution of respiratory symptoms (52, 53). Studies have shown a prevalence of 30 to 82% of stool positivity after viral respiratory clearance (22, 54, 55). Cheung et al. noted a higher median fecal viral load in patients with positive stool viral RNA as compared to individuals without diarrhea (5.1 log_10_ copies/ml vs. 3.9 log_10_ copies/ml; *p* = 0.06) (32). These factors play a significant role in the development of mitigation strategies and standard protocol before a patient could be deemed non-infective after discharge from the hospital.

## COVID-19 and Gastrointestinal Endoscopies

Endoscopic procedures can increase the exposure of the endoscopy staff with spillage of GI secretions, especially with the use of multiple devices (56, 57). Due to the inherent nature of the procedures with proximity to oral-pharyngeal secretions, endoscopy staff can get exposed to increasing the risk of transmission (56). Furthermore, endoscopes such as duodenoscopes are at risk of microbial contamination due to their inherent design (elevator) (58). As endoscopy centers resume their workflow, significant changes in triaging have been implemented with pre-procedural testing and active screening for COVID-19 symptoms. In addition to classical symptoms of fever, cough, shortness of breath, altered taste, additional GI symptoms of nausea, vomiting, diarrhea, and abdominal pain should be a part of a pre-operative questionnaire for elective procedures for those centers in high prevalence areas (59). Multiple GI societies have recommended guidelines for the use of negative pressure rooms, especially for patients infected with SARS-CoV-2 (60, 61). During the procedure, endoscopists, and staff should take adequate precautions such as the use of personal protective equipment (PPE) to prevent transmission of infection. Additionally, if the disinfection of endoscopic equipment is inadequate, it can theoretically lead to contamination and spread (58, 62). Repici et al. reported data to assess the risk of COVID-19 transmission in GI endoscopy (63). A study composed of 851 patients from Northern Italy showed eight patients had symptoms of fever, cough of which only one patient turned COVID-19 positive. None of the patient required hospitalization, suggests a very low risk of endoscopic transmission SARS-CoV-2 for patients. Furthermore, Repici et al. assessed 968 health care workers (HCW) from 41 hospitals, 42 (4.3%) tested positive, and six (0.6%) were hospitalized (63). Of the 42 HCWs who tested positive, 85.7% occurred prior to the introduction of PPE or reduction of endoscopy volume. All of these point toward the low risk of transmission of SARS-CoV-2 during endoscopy (63). Nevertheless, endoscopy staff should adhere to strict protective measures to avoid any amount of transmission.

Multiple national and international societies recommended deferral of non-urgent and elective procedures during the “phase 1” of the pandemic. This led to significant changes in the functioning of endoscopy units. In countries like Brazil, endoscopy staff has been divided into COVID and non-COVID teams to facilitate the flow in the unit (64). Mask mandates have been issued for all the endoscopy staff (56, 57, 61). Layouts of endoscopy units were changed based on risk-based color-coding of the suite, waiting, and recovery rooms (65) Pre-procedure testing has been implemented across multiple endoscopy units (66). Studies showed a reduction of procedure volume up to 99% (67). Studies showed that these changes have led to a decrease in colon cancer screenings by almost 85% (68). Deferral of these procedures was predominately elective (such as screening, surveillance), resulting in potentially increased load during recovery or “phase 2.” Although this is dependent on the rate of infectivity in the community and indication, it is likely expected that increased case volumes and backlogs will occur post-pandemic.

## Medications and GI Symptoms

Patients with COVID-19 are subjected to increased pharmacological interventions. Due to suspicion for secondary bacterial infections, they are empirically treated with antibiotics such as fluoroquinolones and cephalosporins. Antiviral agents such as ritonavir-lopinavir, hydroxychloroquine, remdesivir, and tocilizumab can potentially cause nausea, vomiting, and diarrhea (69–72). Other agents such as azithromycin, oseltamivir, favipiravir may be used in COVID-19 patients at different stages of the disease, which can all contribute to the GI symptoms. If these agents directly cause the GI symptoms or contribute to the cytopathic effects of the SARS-CoV-2 is unclear. Recently, the use of PPI and resulting hypochlorhydria being recognized as a potential for increased positivity of COVID-19. A recent retrospective study showed that pre-hospitalization PPI exposure in hospitalized COVID-19 patients associated with a higher risk of the need for mechanical ventilation and higher mortality (19, 20, 73, 74). Gastric acid has shown to have neutralizing effects on many bacteria and viruses. The similar effect of gastric acid is also proposed on its neutralizing effect on SAR-CoV-2 PPI ca cause profound hypochlorhydria, which could be the reason for the higher risk of COVID-19 in these patients. However, a similar effect was not observed for the H2blockers, which are weak acid-suppressing medications. Further studies are required to discern if PPI use increases the viral stools shedding in COVID-19.

## Conclusion

GI manifestations are increasingly being recognized in COVID-19 patients. Some studies have shown severe disease in these patients, which could be due to increased viral load and involvement of multiple organ systems. It is important to recognize that some patients with COVI-19 may have only GI symptoms either prior to or in the absence of subsequent respiratory symptoms. These symptoms can be varied in presentation–from loss of sense of taste and smell to severe GI upset with diarrhea and abdominal pain. Individuals with these symptoms working in healthcare or other higher-risk environments should be checked for Covi-19 and potentially isolates. A strict medical definition of diarrhea should be observed in these patients to differentiate if the virus itself directly causing diarrhea or it is due to the patients' overall sickness. Other potential causes of diarrhea, such as clostridium difficile and antibiotics-associated diarrhea, need to be ruled out in these patients. The role of viral stool positivity in the transmission of the COVID-19 needs to be further studied. Multiple studies have shown that endoscopy staff is at higher risk of acquiring SARS-CoV-2 infection, possibly because of aerosolization of the secretions during suctioning. As endoscopies procedures are being resumed, strict adherence to universal precautions and use of personal protective equipment is needed. The patient viral transmission during the endoscopic procedures has not been reported but is theoretically possible as there are reports viral transmission with other kinds of viruses.

There is an urgent need for the standardization of stool testing, disease severity, a strict definition of GI symptoms, and evaluation of potential confounders. Nevertheless, advances made so far have increased our understanding of the GI symptoms, and they will likely continue to evolve as this pandemic unfolds.

## Author Contributions

HG and AP: conception, design, and literature review. AP: first draft. All authors: critical revision, editing, and final approval.

## Conflict of Interest

The authors declare that the research was conducted in the absence of any commercial or financial relationships that could be construed as a potential conflict of interest.
